# Targeting the tumor microenvironment in pediatric gliomas: Advances and future directions in immunotherapy

**DOI:** 10.1093/noajnl/vdaf193

**Published:** 2025-09-01

**Authors:** Cheyenne Ahamed, Lam Nguyen, Cayley S Brock, Ayla Farzamnia, Pierrick Millet, Keisaku Sato, Kevin K Kumar

**Affiliations:** Deparment of Neurosurgery, The University of Texas at Austin, Dell Medical School, Austin, TX, USA; Deparment of Neurosurgery, The University of Texas at Austin, Dell Medical School, Austin, TX, USA; Deparment of Neurosurgery, The University of Texas at Austin, Dell Medical School, Austin, TX, USA; Deparment of Neurosurgery, The University of Texas at Austin, Dell Medical School, Austin, TX, USA; Deparment of Neurosurgery, The University of Texas at Austin, Dell Medical School, Austin, TX, USA; Deparment of Neurosurgery, The University of Texas at Austin, Dell Medical School, Austin, TX, USA; UT Health Austin Pediatric Neurosciences, Dell Children’s Medical Center, Austin, TX, USA; Deparment of Neurosurgery, The University of Texas at Austin, Dell Medical School, Austin, TX, USA

**Keywords:** computational modeling, immunotherapy, pediatric glioma, tumor microenvironment

## Abstract

The tumor microenvironment (TME) is a critical determinant of tumor progression and therapeutic response in gliomas. While pediatric gliomas have historically been treated using strategies derived from the management of adult gliomas, emerging evidence reveals that pediatric gliomas possess a unique TME. The pediatric TME is distinct, characterized not only by differences in cellular composition but a lower mutational burden, diminished neoantigen presentation, and heightened immunosuppressive activity. The unique immune landscape, developmental trajectories, and immune escape mechanisms in the pediatric TME create barriers to effective therapy. Recent studies show promising results in novel and advanced therapeutic strategies, highlighting the potential for innovative immunotherapeutic approaches. Advances in methodologies for modeling the TME, including computational approaches and animal-based models, provide new insights into pediatric glioma biology. Utilization of computational models may provide opportunities to predict tumor response to specific therapies and tailor immunotherapy regimes to individuals, allowing for personalized care. Leveraging the unique features of the pediatric TME offers an opportunity to overcome current barriers to immunotherapy and develop more effective, age- and tumor-specific treatment strategies.

**Key Points**

- The tumor microenvironment of pediatric gliomas differs significantly from adults.
- Lower mutational burden, reduced neoantigen presentation, and heightened immunosuppression hinder therapy.
- Emerging immunotherapies and advanced tumor modeling can help better target pediatric glioma.

Brain tumors, accounting for approximately 25% of all pediatric cancers, are the leading cause of cancer-related deaths in children in developed countries.^1^ Pediatric gliomas are the most common type of CNS tumor in children and are classified as either low-grade or high-grade based on histological features.^2^ Patients with high-grade gliomas typically have poor prognosis compared to their low-grade counterparts, with a median survival of about one year.^2^

Pediatric gliomas exhibit considerable molecular heterogeneity that contributes to their diverse tumor microenvironment (TME) and variable responses to immune-based therapies.^3^ The 2021 WHO Classification incorporated molecular profiling into diagnostic criteria, allowing for categorization based on specific genetic alterations.^4^ This has allowed for both pediatric low-grade gliomas (pLGGs) and pediatric high-grade gliomas (pHGGs) to be stratified into distinct molecular subgroups, defined by their genetic drivers. pLGGs such as pilocytic astrocytomas and gangliogliomas are frequently driven by activation of the RAS-MAPK pathway.^5^ Approximately 35% of pLGGs harbor structural variants such as KIAA1549::BRAF fusions, while another 20% possess single-nucleotide variants, most commonly the BRAF V600E point mutation.^5^ Additional cases involve both structural and single-nucleotide variants in FGFR1/2/3.^5^ pLGGs also often involve a single genetic aberration and are associated with a favorable prognosis, separating them from their adult counterpart, which exhibit a higher mutational burden and increased likelihood of malignant transformation.^6^ In contrast, pHGGs are more genetically diverse.^6^ Infant hemispheric gliomas often contain NTRK fusions and other receptor tyrosine kinase (RTK) alterations, including ALK, ROS1, and MET fusions.^6^ Diffuse midline gliomas (DMG) are defined by H3K27M mutations, with H3.1 variants typically located in the pons and H3.3 variants more frequent in thalamic, spinal, and cerebellar locations.^6^ Diffuse hemispheric gliomas (DHG) are enriched for H3.3 G34 mutations, while histone- and IDH-wild type pHGGs often show BRAF mutations, CDK deletions, and RTK aberrations.^6^

Traditional treatments, including surgical resection, radiation, and chemotherapy, have varying efficacy across tumor subtypes.^7^ Gross total resection shows a high survival benefit but safely achieving it may be challenging due to tumor location and infiltration.^8^ While radiotherapy is one of the most effective nonsurgical treatments in pediatric glioma, recurrence is commonly observed due to acquisition of radioresistance.^9^ It also risks long-term effects, including secondary malignancy and cognitive impairments.^9^ Children treated with chemotherapy following surgical resection or radiotherapy have significantly better outcomes and recent trends advocate for early chemotherapy use.^10^ However, differences in molecular profiles within tumor types lead to variable outcomes, raising the risk of exposing pediatric patients to additional toxicity without major benefit.^11^

The tumor microenvironment (TME) consists of cells, blood vessels, and molecules within the surroundings of a tumor that can both positively or negatively affect tumor growth.^12^ A tumor and its TME exert reciprocal effects on each other; while a tumor relies on the TME for support, the TME also influences how the tumor develops and grows.^13^ The TME of pediatric tumors typically displays a lower mutational burden and limited expression of mutation-derived antigens that reduces immunogenicity, allowing these tumors to evade immune surveillance and develop resistance to immunotherapy.^14^

Immunotherapy offers a targeted alternative to conventional treatments by leveraging the precision of the immune system to coordinate a focused response to cancer cells.^15^ This approach minimizes the toxicity with traditional therapies, making it a promising option for pediatric patients, where maintenance of neurodevelopmental performance is paramount.^15^ Unfortunately, immunotherapy for pediatric gliomas has yet to demonstrate as robust therapeutic efficacy in comparison to adult gliomas.^16^

One major barrier to immunotherapy efficacy is the immaturity of the pediatric immune system due to its reliance on innate, non-specific immunity and the underdevelopment of adaptive, antigen-specific responses.^17^ This immaturity manifests in distinct histological and molecular features that set pediatric tumors apart from those in adults.^14^ Another significant limitation is the scarcity of clinical trials focused specifically on this population and the unique features of the pediatric TME.^18^ To address this gap, modeled representations of the pediatric TME are utilized to better explain its components and effect on immunotherapy outcomes.^15^ Recent advancements in mathematical, computational, and novel animal models allow researchers to simulate the pediatric TME, including its unique cellular composition and intracellular interactions.^19^ By incorporating the distinctive features of the pediatric TME into these models, responses to both current and novel immunotherapies can be predicted and subsequently validated with clinical data.^19,20^

This review explores the distinctive features of the pediatric glioma TME and how they differ from adult cases, with implications for developing pediatric-specific immunotherapies. We assess the current state of immunotherapy and highlight modeling approaches that deepen our understanding of the pediatric TME. Our goal is to inform future clinical trials and experimental strategies, ultimately guiding treatment and improving outcomes for children with glioma. The 2021 WHO CNS tumor classification subsumes “pediatric glioblastoma” under molecularly defined entities like diffuse pediatric-type high-grade glioma.^21^ We use the historical term when referring to pre-2021 literature.

## Comparing the Pediatric and Adult TME

The innate immune system plays a crucial role as the first line of response against tumor cells and impacting the adaptive immune response to restrict glioma progression.^22^ Tumor cell proliferation and genetic instability can release damage-associated molecular patterns (DAMPs), which activate innate immune system pathways, prompting efforts to harness these pathways for therapeutic purposes.^22^ One key target is the cGAS-STING pathway, wherein cyclic GMP-AMP synthase (cGAS) detects cytoplasmic double-stranded DNA, a marker of genomic instability, and activates the stimulator of interferon genes (STING).^23^ This leads to the secretion of type I interferons, pro-inflammatory cytokines, and induction of autophagy.^23^ In mouse models of pHGGs, activating the cGAS-STING pathway and adding a STING agonist to radiotherapy significantly improved survival over radiotherapy alone.^24^ Adult glioblastoma mouse models treated with a STING agonist led to decreases in immunosuppressive markers, enhanced myeloid cell activation, and increased numbers of infiltrating CD8+ T and NK cells, demonstrating an ability to alter the TME to a more immunostimulatory phenotype.^25^

Toll-like receptors (TLRs) also respond to DAMPs, supporting immune surveillance, DNA repair, and activation of inflammatory pathways like NF-κB and MAPK.^23^ TLR agonists are being explored for glioma therapy.^26^ The TLR7 agonist imiquimod induces redistribution of CD4 + and CD8 + T cells from peripheral blood into the brain, reduces intratumoral Treg levels, and activates brain-infiltrating lymphocytes.^27^ The TLR9 agonist CpG increases effector T cells relative to Tregs and can eliminate murine intracranial glioma when combined with tumor lysate and effector T cells.^27^ However, high TLR4 expression in glioma cell lines makes it a challenging target, as its activation promotes tumor survival and immune evasion.^27^ Co-activation of the Fas pathway alongside TLR4 agonists has been shown to counteract TLR4’s tumor-promoting effects, suggesting a potential strategy to overcome this challenge.^27^

**Figure 1.**
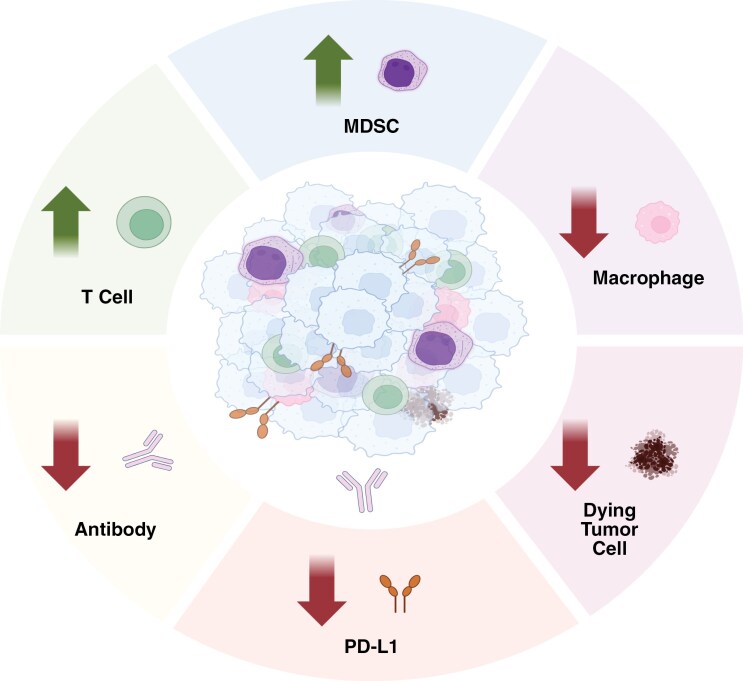
**Proposed Properties of the Pediatric Glioma TME.** Pediatric glioma TMEs are characterized by an increased presence of T cells and myeloid-derived suppressor cells (MDSCs), coupled with decreased levels of PD-L1, macrophage, antibodies, and apoptotic (dying) tumor cells compared to adult TMEs. These differences contribute to the classification of pediatric tumors as predominantly “cold,” indicating lower immune activity, while adult tumors are often described as “hot,” reflecting higher immune activity. Created in BioRender. Kumar, K. (2025) https://BioRender.com/ogbpxfz.

The tumor microenvironment (TME) greatly affects immunotherapy efficacy, and its complexity and heterogeneity make targeting pediatric gliomas especially challenging.^28,29^ Pediatric gliomas are classified as “cold” tumors from an immunological perspective, characterized by a low mutational burden and a lack of neoantigens.^30^ This paucity of neoantigens makes mounting an immune response challenging as it prevents the activation of lymphocytes, particularly cytotoxic T lymphocytes (CTLs).^30^ The TME consists of several distinct cell types, including myeloid-derived suppressor cells (MDSCs), eosinophils, neutrophils, and T cells (**Figure 1**).^9^

Both the diversity of cell types within the TME and the relative proportions of each type have implications for tumorigenicity and metastasis.^31^ Populations of tumor-associated macrophages (TAMs) derived from the TME are either brain-resident microglia or bone marrow-derived macrophages.^32^ In adult glioblastomas, infiltrating macrophages account for 85% of the TAM population, while resident microglia account for the remaining 15%.^33^ TAMs contribute to the immunosuppressive microenvironment by releasing macrophage-derived cytokines that lead to immune evasion and result in tumor progression.^34^ Macrophage polarization is influenced by the molecular components of its surrounding environment dictating whether the macrophage adopts a classically activated phenotype or an alternatively activated phenotype. Classically activated macrophages express pro-inflammatory cytokines such as interleukin (IL)-12, IL-23, and tumor necrosis factor alpha (TNFα), while alternatively activated macrophages express anti-inflammatory molecules, such as IL-10 and transforming growth factor beta (TGF-β).^35–37^ Classically activated macrophages are considered to be involved in the anti-tumor response, adopting this phenotype in response to interferon-γ released by innate and adaptive immune cells.^38^ They produce pro-inflammatory cytokines implicated in killing tumor cells, recruit immune cells, and create an inflammatory TME that hinders the growth and spread of tumor cells.^38^ Conversely, alternatively activated macrophages are considered pro-tumorigenic by promoting angiogenesis, tumor remodeling and an anti-inflammatory TME that allows for immune cell evasion and tumor growth.^39^

In adult glioblastomas, the TME is dominated by alternatively activated macrophages, shifting the balance toward immunosuppression.^40^ These macrophages prevent immune cell recruitment, express high levels of programmed cell death ligand 1 (PD-L1) to suppress T cell activation, and release factors like vascular endothelial growth factor and platelet-derived growth factor to promote angiogenesis and tumor invasion.^41,42^ However, studies indicate that TAMs function differently in pediatric gliomas than in adults. For instance, although high TAM expression is associated with shorter overall survival in adult glioblastomas, this correlation is not observed in pediatric cases.^43^

Pediatric gliomas exhibit fewer immune cells overall, including a notably lower abundance of T cells compared to their adult counterparts.^44^ T-cell infiltration is limited in pediatric gliomas, with T cells clustering in perivascular spaces, rather than infiltrating deeper into the tumor parenchyma.^45^ Studies in adult glioblastomas have shown an increased presence of CD4 + CD56 + T cells, suggesting an immunoregulatory role that may contribute to immune evasion.^46^ Pediatric gliomas also demonstrate higher varieties in T-cell populations depending on tumor subtypes when compared to adult gliomas.^47^ Higher T-cell counts were found in pLGGs than in pHGGs with both exhibiting tissue-resident memory T cells with CD103 + and T-cell factor 1 + subpopulations that have distinct spatial localization.^48^ pHGGs and pLGGs show higher expression of CD8 + T cells, whereas DMGs exhibit minimal immune cell infiltration and low inflammation.^48^ Subtype differences extend to TAM populations as well with the number of CD163 + macrophages markedly increased in pLGGs and pHGGs but not in DMGs and the ratio of CD163 + to CD68 + macrophages elevated with no elevation found in DMGs.^49^

Reduced expression of PD-L1 is observed in pediatric gliomas, suggesting that pediatric gliomas may utilize different immune evasion techniques than adults.^50^ The binding of PD-L1 to its receptor programmed cell death protein 1 (PD-1) is an important feature of immune evasion for tumor cells, as it leads to T-cell disruption and apoptosis, allowing for tumor cells to escape detection from the immune system.^51^ In adult gliomas, studies have found PD-L1 expression to be as high as 88% whereas expression in pediatric gliomas averaged to 45%.^52^ Although PD-L1 is rarely expressed on tumor cells in pediatric gliomas, its correlation with TAMs suggests it may still represent a viable immunotherapeutic target.^53^ MDSC cells contribute to creating an immunosuppressive TME by presenting PD-L1 to T-cells, polarizing macrophages to alternative activation, and promoting tumor growth through enhanced angiogenesis.^54^ High levels of MDSC infiltration are present in both pediatric DMG and adult glioblastomas, making it a potential therapeutic target.^55^

pHGGs have been found to express distinct epigenetic signatures from their adult counterparts. Pediatric DMGs typically harbor histone H1 and H3 mutations, with a K27M mutation frequently found as well as H3.3 G34 mutations.^56^ H3 mutations are found in over 50% of pHGGs but only less than 0.2% of adult HGGs.^57^ These genetic differences may drive the occurrence of an immunologically cold microenvironment, as demonstrated by K27M’s impact on lowering immune cell infiltration.^56,58^ The limited efficacy of immunotherapy in pediatric gliomas are partly explained by differences between potential immunotherapy targets, secondary to variations in the TME.^59^ Several immunotherapy trials are underway investigating epidermal growth factor receptor (EGFR) as a target, given that amplification of EGFR is present in 60% of adult HGG; however, these trials have not demonstrated success to date.^55^ In contrast, EGFR amplifications are only observed in 5.5% of pediatric glioblastoma, making it a less viable target and necessitating a reevaluation of immunotherapeutic strategy for pediatric patients.^60^

Single-cell RNA sequencing (scRNA-seq) and expression analysis identified four types of malignant cells within pediatric glioblastomas: neural progenitor cell-like (NPC-like), oligodendrocyte progenitor cell-like (OPC-like), astrocyte-like (AC-like), and mesenchymal-like (MES-like).^61^ Pediatric glioblastomas predominantly exhibit NPC-like and OPC-like states which are highly proliferative, contributing to rapid tumor growth.^61^ In contrast, adult glioblastomas feature more stable, fixed states such as AC-like cells, which are less common in pediatric cases.^61^ These findings suggest that pediatric and adult gliomas differ fundamentally in their origins, with pediatric gliomas rooted in disruptions during development while adult gliomas arise from accumulated genetic mutations over time.^62^ The developing pons and forebrain have unique progenitor and differentiation pathways disrupted in pediatric gliomas.^63^ Additionally, pediatric glioma cells have been linked to specific developmental lineages, with tumors mirroring normal differentiation patterns within their respective lineages.^64^ Diffuse hemispheric gliomas with a driver mutation at glycine 34 in histone 3 (DHG-H3G34) account for over 30% of pHGGs.^65^ Genomic profiling of DHG-H3G34 revealed *TP53* and *ATRX* mutations in over 90% of cases, along with high rates of alterations in cell-cycle regulators such as CDK4 and CDK6 amplification and *PDGFRA* alterations, presenting potential vulnerabilities to CDK4/6 inhibitors and *PDGFRA*-targeted therapy.^66^ Molecular and epigenomic analyses of DHG-H3G34 have identified interneuron progenitors as a potential cell of origin.^67^ These tumors also recapitulate transcriptional and developmental features of GABAergic interneuron lineages.^65^ DHG-H3G34’s developmental program reveals a vulnerability, allowing CDK6 and interneuron differentiation regulators to target its progenitor-like state.^65^ These differences between the pediatric and adult TME underscore the heterogeneity of gliomas and highlight the need for age-specific therapeutic strategies.^62^

## Recent Advancements in Immunotherapy

While immunotherapy has shown promise in adult gliomas, its application in pediatric gliomas remains limited due to their unique features, including a low mutational burden, distinct epigenetic drivers, and an immunosuppressive tumor microenvironment.^30^ Logistical barriers, including few eligible patients and limited trial access near homes, further hinder pediatric brain tumor immunotherapy development.^68^ By examining a range of immunotherapy strategies, we aim to identify gaps shaped by the unique characteristics of pediatric tumors and to highlight areas requiring further investigation and improvement.

### Immune Checkpoint Inhibitors

Immune checkpoint inhibitors (ICIs) have emerged as an innovative form of immunotherapy that targets molecules involved in lymphocyte regulation.^69–71^ The main three that have been extensively studied are cytotoxic T-lymphocyte-associated protein 4 (CTLA-4), PD-1 (CD279), and PD-L1 (CD274).^69–71^ CTLA-4 is expressed on T cells, and it binds to the B7 protein on antigen-presenting cells (APCs) to inhibit lymphocyte activation and differentiation through competitive inhibition of CD28, a stimulatory molecule.^69,72^ The PD-1/PD-L1 pathway suppresses naive T-cell proliferation and differentiation by inhibiting protein kinase signaling pathways.^70,71^ Inhibiting these checkpoints can activate lymphocytes, providing a promising approach for tumor treatment.^72^

A recent retrospective study evaluated pembrolizumab (PBL), an anti-PD-1 monoclonal antibody, in both adult and pediatric brain tumors. However, no patients showed responses mediated by local lymphocytes. This may be due to the large molecular size of PBL (146 kDa), its poor penetration of the blood–brain barrier, and limited T-cell infiltration.^73^ A subsequent study found that anti-PD-1 inhibitors improved survival in pediatric patients with relapsed hypermutant tumors associated with germline DNA repair deficiencies.^70^ Continued use of ICIs resulted in a median survival of 2 years.^74^ Nivolumab, an anti-PD-L1 inhibitor, also demonstrated benefits in pediatric cancers with high mutation burden or mismatch repair deficiency, particularly in tumors microsatellite instability.^71^ Although few studies have examined the effects of ipilimumab, a CTLA-4 inhibitor, alone in pediatric gliomas, reports indicate that neither nivolumab alone nor in combination with ipilimumab improved survival in pediatric patients with CNS tumors.^75^

More recent immune checkpoint inhibitors under investigation in pediatric gliomas target T-cell immunoglobulin and mucin-domain-containing-3 (TIM-3) and indoleamine 2,3-dioxygenase (IDO).^76–78^ TIM-3 is an inhibitory molecule on the surfaces of immune cells and a significant component of the TME in DMG.^75^ TIM-3 inhibition in syngeneic mouse models prevents DMG recurrence by promoting TME inflammation and T-cell activation.^76^ IDO controls inflammation and T-cell immune tolerance by metabolizing tryptophan into kynurenine.^77^ In a recent phase I trial, pediatric brain tumor patients received indoximod, an indoleamine 2,3-dioxygenase 1 inhibitor, alongside chemotherapy and radiation.^78^ Indoximod therapy was well tolerated, and responders had a median overall survival three times longer than nonresponders.^78^ Immune checkpoint inhibitors have shown limited efficacy in pediatric gliomas, largely due to key challenges such as low mutational burden, which reduces neoantigen presentation and impairs immune recognition.^30^ The glioma TME is highly immunosuppressive, with fewer infiltrating B and T cells, limiting targets for checkpoint inhibitors and reducing ICI efficacy in pediatric gliomas.^72^

### CAR T-Cell Therapy

Chimeric antigen receptor (CAR) T-cell therapy, a specialized form of adoptive cell therapy (ACT) in which T cells are genetically modified to target cancer, has emerged as a promising new immunotherapy for pediatric glioma.^79,80^ CAR T cells activate T lymphocytes through direct antigen recognition, bypassing major histocompatibility complex (MHC)-mediated antigen presentation.^80^ They consist of three components: an extracellular region for antigen recognition, a transmembrane region for stability, and an intracellular region for signal transmission into T cells.^80^ The affinity of the extracellular antigen-binding domain has been modulated to improve CAR specificity and reduce “on-target, off-tumor” side effects.^81^ Modifications to transmembrane domains (TMDs), such as incorporating native TMDs such as NKG2D and developing multi-chain interactions, have improved CAR stability and anti-tumor potency by altering dimerization and intracellular signaling.^81^ Immunoreceptor tyrosine activation motifs (ITAMs) are phosphorylation sites on cytoplasmic domains and are central to CAR design, with their number and diversity influencing CAR modification.^81^ CAR T cells can be delivered intravenously, intrathecally, or intracerebroventricularly.^82^

Interest in CAR T therapy for brain tumors emerged from ACT studies in melanoma brain metastases, showing patient-derived immunotherapies can be effective in neuro-oncology.^83^ Subsequent studies have identified key biomarkers for CAR T therapy in pediatric brain tumors.^84–86^ Analysis of patient-derived orthotopic xenograft samples identified a hierarchy of biomarker expression in pediatric tumor cells, with B7 homolog 3 (B7-H3), an immunosuppressive signaling protein, and disialoganglioside (GD2), a molecule that facilitated tumor proliferation, showing the highest levels.^84^ CAR T cells targeting B7-H3 demonstrated significant anti-tumor activity.^84^ Subsequent studies of high-grade pediatric gliomas have demonstrated the significant anti-tumor effect of B7-H3 CAR T cells in immunocompetent xenograft models.^85,86^

GD2 is another prevalent antigen target that is highly expressed on glioma cells with the histone H3K27M mutation.^87^ CAR T cells against the GD2 antigen induced clinical and radiographic improvement in the majority of patients trialed with H3K27M-mutated DMG or spinal cord DMG.^88^ Toxicities associated with GD2-CAR T cells were tolerable and reversible with supportive treatment.^88^ Similarly, human epidermal growth factor receptor 2 (HER2)-targeting CAR T cells have shown efficacy, demonstrating antigen-specific cytotoxicity and tumor regression in DMG xenograft models.^89,90^

Despite its promise, CAR T-cell therapy faces notable challenges. Nonspecific-targeting of CAR T cells against normal cells can lead to significant adverse effects, such as cytokine release syndrome, immune effector cell-associated neurotoxicity syndrome (iCANS), and tumor inflammation-associated neurotoxicity (TIAN).^91^ Intraventricular administration of CAR T cells is associated with a lower incidence and severity of these adverse effects.^82^ Antigen heterogeneity and the suppressive tumor microenvironment found in glioma cells can also impede CAR T cell trafficking and efficacy a phenomenon observed using CAR T cells against epidermal growth factor receptor variant III (EGFRvIII) in adult glioma cells.^79^ Analysis of brain tumor samples after administration demonstrates a wide variety in the expression of EGFRvIII along with immunosuppressive molecules, such as IDO1, PD-L1, and IL-10, from the tumor microenvironment.^92^ Antigenic heterogeneity within the same tumor cells often results in antigen escape and lessens the efficacy of CAR T-cell therapy.^93^ Current efforts aim to develop CAR T-cell therapies that target multiple antigens simultaneously to overcome tumor heterogeneity and reduce the risk of antigen escape. Development of a trivalent T-cell product containing CAR molecules targeting HER2, IL13Rα2, and EphA2, helped overcome interpatient antigenic variability glioblastoma sample cohort.^93^ Clinical trial (NCT05168423) developed a bivalent CAR T cell targeting both EGFR and IL13Rα2.^94,95^ Early results showed minimal neurotoxicity at two doses of CART-EGFR-IL13Rα2 cells, with limited cases of iCANS.^94^

Updated data demonstrated that the majority of patients experienced tumor regression, indicating that CART-EGFR-IL13Rα2 cells have significant anti-tumor effects.^95^ NCT05660369 using CARv3-TEAM-E cells, which target EGFRvIII via a second-generation CAR T cell while secreting antibodies against wild-type EGFR.^96^ In three patients, the treatment showed no grade 3 + adverse effects and achieved rapid radiographic tumor regression within days, though responses were transient in two patients.^96^ The BrainChild-04 study (NCT05768880) is the first clinical trial using CAR T cells to target multiple antigens in pediatric CNS tumors is currently underway.^97^ Ongoing or recently completed clinical trials of CAR T cells pediatric brain tumors are targeting antigens such as EGFR806, HER2, GD2, and B7-H3 (**Table 1**).

**Table 1.** Relevant CAR-T Cell Clinical Trials for Pediatric Gliomas

NCT Number	Phase	Targets	Number of Patients	Glioma Type	Administration Route: Intravenous (IV), Intrathecal (IT), or Intracerebroventricular (ICV)	Status	Reference
NCT03638167	1	EGFR806	11	recurrent or refractory EGFR-positive CNS tumors	ICV	Recruiting	Rao et al., 2022^89^
NCT03500991	1	HER2	10	recurrent or refractory HER2-positive CNS tumors	ICV	Active but not recruiting	Rao et al., 2022^89^
NCT04185038	1	B7-H3	90	DMG and B7-H3-positive refractory CNS tumors	ICV	Recruiting	Lin et al., 2022^79^
NCT04099797	1	GD2	37	GD2-positive DMG & HGG, medulloblastoma	ICV	Recruiting	Lin et al., 2022^79^
NCT05168423	1	EGFR, IL13Ra2	13	Adult recurrent glioblastoma	IT	Recruiting	Bagley et al., 2025^94^
NCT05660369	1	WT-EGFR, EGFRvIII	3	Adult recurrent glioblastoma	ICV	Recruiting	Choi et al. 2024^96^
NCT05768880	1	B7-H3, EGFR806, HER2, IL13-Zetakine	72	DMG and recurrent or refractory CNS tumors	ICV	Recruiting	

### Tumor Neoantigen Vaccine

The evolution of immunotherapy has occurred alongside the continued development of vaccines.^98^ Cancer vaccines induce immunological memory by exposing immune cells, specifically CD8 + T cells, to tumor-associated antigens.^98–100^ Two main vaccine types explored and tested for the treatment of pediatric gliomas are dendritic cell (DC) and peptide vaccines (**Table 2**).^99,100^

**Table 2.** Relevant Tumor Neoantigen Vaccine Clinical Trials for Pediatric Gliomas

NCT Number	Phase	Vaccine type	Number of Patients	Glioma Type	Status	Reference
NCT02840123	1	Dendritic Cell	9	DMG	Unknown	Benitez-Ribas et al., 2018^101^
NCT01130077	1	Peptide	26	Brainstem and non-brainstem pHGGs	Completed	Pollack et al., 2014^102^
NCT04808245	1	Peptide	15	H3K27M + DMG	Recruiting	Grassl et al., 2023^103^
NCT04573140	1 & 2	Nucleic Acid (mRNA)	52	pHGG & adult glioblastoma	Recruiting	Mendez-Gomez et al., 2024^104^

Dendritic cells are antigen-presenting cells (APCs) that bridge innate and adaptive immunity by processing and presenting antigens to immune cells such as CD8 + and CD4 + T cells.^98^ In dendritic vaccines, autologous DCs are isolated via apheresis, matured, loaded with tumor-specific antigens, and administered to patients.^98^ These cells induce cytokine release and T-cell activation.^98,99^ A phase Ib study of autologous dendritic cell vaccines (ADCV) in nine children with newly diagnosed diffuse intrinsic pontine glioma (DIPG) after radiation therapy showed the vaccine was feasible, safe, and induced DIPG-specific immune responses in peripheral blood and cerebrospinal fluid.^100^ These findings support ADCV as a promising option for future immunotherapies.^100^ Furthermore, the cytomegalovirus (CMV) antigen pp65 in pediatric medulloblastoma and malignant glioma has received significant research interest.^98^ A Phase I trial (NCT03299309) evaluated the safety of the peptide (PEP)-CMV dendritic vaccine, targeting the CMV pp65 antigen in children and young adults with recurrent malignant gliomas and medulloblastomas.^98^ Among 22 patients, the vaccine demonstrated no severe toxicities, elicited an immune response in 75% of patients, and showed stable disease or partial response in 6 of 11 evaluable cases.^98^

Peptide vaccines directly introduce tumor-associated antigens into circulation, allowing endogenous APCs to process them and activate lymphocytes.^98^ In a phase I trial (NCT01130077), emulsified peptide epitopes of glioma-associated antigens, such as EphA2, interleukin-13 receptor alpha 2 (IL-13Rα2), and survivin, were administered to 26 pediatric patients with newly diagnosed brainstem gliomas or pHGGs.^102^ Five patients experienced pseudoprogression, which resolved with dexamethasone.^102^ 19 patients had stable disease, two showed partial responses, two had minor responses, and two remained disease-free for an extended period following surgery.^102^

A significant discovery within peptide vaccine research is the H3K27M mutation, a point mutation/neoepitope in histone 3 that is highly prevalent in DMG.^105^ A H3K27M peptide vaccine elicited robust cytotoxic T-cell and T-helper-1-cell-driven interferon gamma immune responses, indicating the peptide was properly processed and displayed by APCs.^105^ A phase I clinical trial has tested this vaccine in adult patients with H3K27M + DMG and reported it to be safe, tolerable, and immunogenic, with radiographic improvement.^103^ Other vaccine approaches include nucleic acid vaccines, which utilize mRNA or plasmid DNA to express tumor antigens through viral recombinant vectors.^98^ A current trial is testing mRNA vaccines against glioblastoma in adults, following canine studies showing RNA lipid particles enhance antigen presentation, interferon signaling, and cytotoxic activity.^104^

Although vaccine development for pediatric gliomas has advanced, several challenges persist. The weak immunogenicity of current tumor vaccines results in inadequate CD8 + T-cell responses. In addition, downregulation of tumor antigens, especially those presented by MHC class I, allows gliomas to evade immune detection.^104^ As a result, glioma vaccines remain largely ineffective against the suppressive tumor microenvironment.

### Oncolytic Virotherapy

Oncolytic virotherapy uses viruses to selectively infiltrate and terminate tumor cells.^101,106^ One of the most studied viruses in the treatment of pediatric gliomas is herpes simplex virus-1 (HSV-1) G207.^101^ Cerebellar inoculation of HSV-1 G207 effectively regressed medulloblastoma tumors without long-term persistence or toxicity.^101^ In clinical trial PNOC020, treatment of pHGGs with the HSV-1 G207 led to a positive therapeutic response and increased infiltration of lymphocytes in tumor cells.^106^ Notably, this therapy strengthened immune response by converting historically immunological silent tumors to more immunogenic ones.^106^

Adenovirus is also highly utilized in oncolytic virotherapy.^107^ DNX-2401, a genetically modified oncolytic adenovirus, demonstrated safety and increased survival rate in both immunodeficient and immunocompetent models of pHGGs and DMG.^107^ In clinical trial NCT03178032, 12 pediatric patients with newly diagnosed DMG received two different doses of DNX-2401 followed by radiotherapy. While the administration of DNX-2401 led to either a reduction in or stabilization of tumor size, patients also reported serious adverse effects such as hemiparesis and tetraparesis.^107^ In the first-in-human and first-in-child trial (NCT01844661), autologous mesenchymal stem cells carrying an oncolytic adenovirus were used in pediatric patients with relapsed or refractory solid tumors.^108^ Repeated infusions caused only mild toxicities, such as fever, and adenovirus replication was confirmed by PCR in most patients.^108^ Tumor stabilization was observed in 2 out 9 participants.^108^

Other oncolytic viruses under investigation include polio virus and reovirus.^109,110^ A major phase 1b clinical trial (NCT03043391) investigated the use of the Sabin-rhinovirus poliovirus (PVSRIPO) for recurrent malignant pediatric gliomas.^109^ Convection enhanced delivery, delivery of therapeutics directly to the brain, of the vaccine was safe and tolerable in subjects.^109^ For reovirus, a phase I trial (NCT02444546) and viral clearance study confirmed that the sargramostim (reovirus) therapy was well-tolerated in pediatric patients with recurrent or refractory high-grade brain tumors with transient changes in immune cells.^110^

While oncolytic virotherapy shows therapeutic potential, several challenges hinder its broader application in pediatric brain tumors. First, the blood–brain barrier significantly limits the delivery of oncolytic viruses, resulting in the need for alternative delivery methods like intratumoral or intracranial injection.^111,112^ Second, the low somatic mutational burden of pediatric tumors results in limited tumor-associated antigens for immune cells to recognize. This ultimately limits the immune-mediated responses triggered by oncolytic viruses.^112^ Finally, antiviral immunity, including neutralizing antibodies and innate immune responses, can eliminate the viruses before they can have therapeutic impact.^113^**Table 3** summarizes oncolytic therapy clinical trials.

**Table 3.** Mentioned Oncolytic Virotherapy Clinical Trials for Pediatric Gliomas

NCT Number	Phase	Virotherapy Type	Number of Patients	Glioma Type	Status	Reference
NCT02457845	1	HSV-1	13	recurrent or progressive pHGG	Completed	Friedman et al., 2021^107^
NCT03178032	1	Adenovirus	12	DMG	Completed	Martínez-Vélez et al. 2022^108^
NCT01844661	2	Adenovirus	20	Metastatic and refractory solid tumors	Completed	Ruano et al. 2020^109^
NCT03043391	1	Poliovirus	8	Recurrent pHGG	Completed	Thompson et al., 2023^110^
NCT02444546	1	Reovirus	6	pHGG	Completed	Schuelke et al., 2022^111^

### Microglial Replacement

Microglia are resident myeloid-derived immune cells of the CNS.^114^ They start colonizing the neuroepithelium early in fetal development.^115,116^ During this early period, precursor microglial subsets with varying morphologies, molecular biomarkers, and function have been identified in different regions of the brain.^115^ Since pediatric brain tumors are thought to arise during prenatal brain development, tumor development can potentially be affected by the immature microglia inhabiting a particular area of the brain.^115,116^ For example, microglia reach peak concentrations in the midline brain, a region commonly associated with pediatric tumor development, such as DMG.^116^

The importance of microglia in pediatric glioma research has been highlighted by the results of several clinical studies.^117–119^ Immature microglia in the cerebellum and corpus callosum exhibit high levels of insulin-like growth factor, promoting the expansion and survival of neural precursor cells in sonic hedgehog (SHH)-subtype medulloblastoma.^117^ Myeloid cells from mature murine SHH medulloblastoma models enhanced tumor cell killing.^117^ This leads to the implication that microglia can assume anti- or pro-tumor phenotypes based on their level of maturation. Microglia can also mimic epigenetic changes found in tumor cells.^118^ Microglia can exhibit the loss of lysine 27 methylation that is typically observed in H3K27M^+^ DMG.^119^

The therapeutic potential of manipulating microglia is demonstrated by the recent development of microglial replacement therapy.^120,121^ This bone marrow transplantation based method replaces microglia with donor circulation-derived myeloid cells (CDMCs) with high efficiency.^120^ Colony-stimulating factor 1 receptor (CSF1R) is essential for microglial survival, leading researchers to use CSF1R inhibitors such as PLX5622 to deplete resident microglia, followed by bone marrow transplantation to achieve effective microglial replacement.^120^ This technique has shown promise in mice with experimental autoimmune encephalomyelitis, conferring a neuroprotective myeloid state, resulting in improvement of neurological deficits and reduced number of demyelinating lesions.^121^

**Figure 2.**
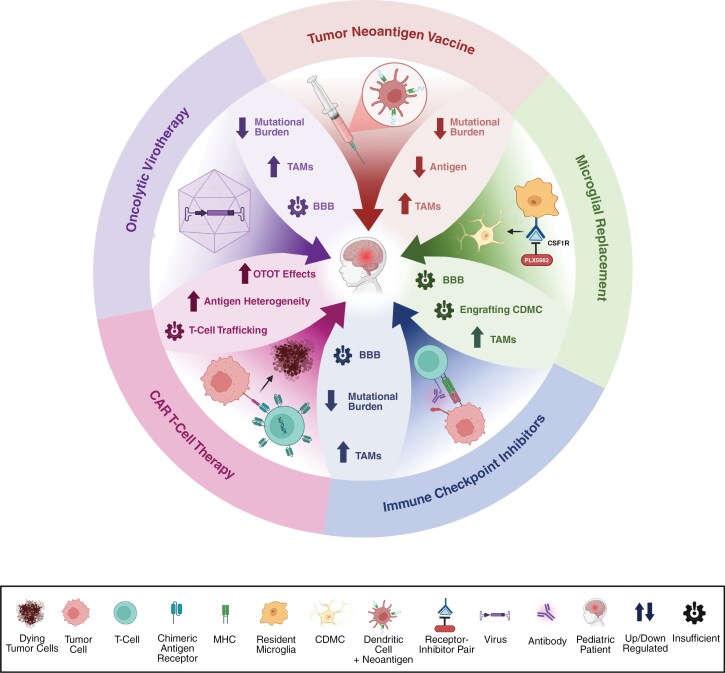
**Challenges in the Application of Immunotherapies for Pediatric Cancer Treatment.** Immunotherapies have revolutionized cancer treatment, but their application in pediatric patients presents unique challenges: *Tumor Neoantigen Vaccines* are limited by a lower mutational burden, reduced neoantigen availability, and elevated levels of tumor-associated macrophages (TAMs). *Microglial Replacement Therapies* face barriers including the blood-brain barrier (BBB), increased TAM infiltration, and difficulty in engrafting circulation-derived myeloid cells (CDMC). Approaches such as PLX5622, a selective CSF1R (colony-stimulating factor 1 receptor) inhibitor, are used to deplete endogenous microglia and facilitate engraftment of CDMCs. *Immune Checkpoint Inhibitors* are constrained by the BBB, low mutational burden, and high TAM prevalence. *Chimeric Antigen Receptor (CAR) T-Cell Therapy* is subject to increased on-target, off-tumor (OTOT) toxicity, antigen heterogeneity, and insufficient T-cell trafficking to tumor sites. *Oncolytic Virotherapy* encounters obstacles such as a low mutational burden, elevated TAM levels, and limited viral delivery across the BBB. This figure summarizes the key biological and physiological challenges that impede the effectiveness of immunotherapies in pediatric cancer patients, highlighting areas for further research. Created in BioRender. Kumar, K. (2025) https://BioRender.com/a8kon4y.

Microglial replacement shows promise for treating pediatric gliomas, but more research is needed to understand their role in CNS malignancies. Studies have demonstrated that tumors can “hijack” the genetic expression of microglial cells and trigger a pro-tumor state.^122^ The variety in functions of these cells have made it difficult to predict their role in the tumor microenvironment.^122^ Studies also suggest that chemotherapy and radiotherapy alter the myeloid cells within the tumor microenvironment.^123^ Furthermore, a recent study reported that triggering receptors expressed on myeloid cell 2 (TREM2), an important signaling molecule for microglial function, is upregulated in glioma-associated microglia and has a negative correlation with overall survival.^124^ Investigating the interaction between signaling molecules like TREM2 and microglia will enhance our understanding of microglial function within the tumor microenvironment. However, key biological and physiological barriers continue to limit the effectiveness of immunotherapies in pediatric cancer patients (**Figure 2**).

## Modeling the TME

The distinct features of the pediatric glioma TME limit the effectiveness of immunotherapies developed for adult tumors, highlighting the need for pediatric-specific approaches.^125^ Direct study of the TME is constrained by the limited number of patients eligible for clinical trials and preclinical testing. To bridge this gap, models are needed that accurately replicate both the tumor and its microenvironment to enable meaningful clinical translation.^126^

Traditional in vitro models identify molecular characteristics and therapeutic targets, but struggle in capturing tumor heterogeneity and the dynamic TME.^127^ In vivo models offer a more complex understanding of tumor growth within a living system, but are resource-intensive and limited in visualizing individual steps and controlling precise variables.^128^ Computational and mathematical modeling serve as a bridge between in vitro and in vivo studies by integrating distinct tumor features and simulating complex interactions within the TME, without time-intensive experiments.^129^

### Discrete Models

**Figure 3.**
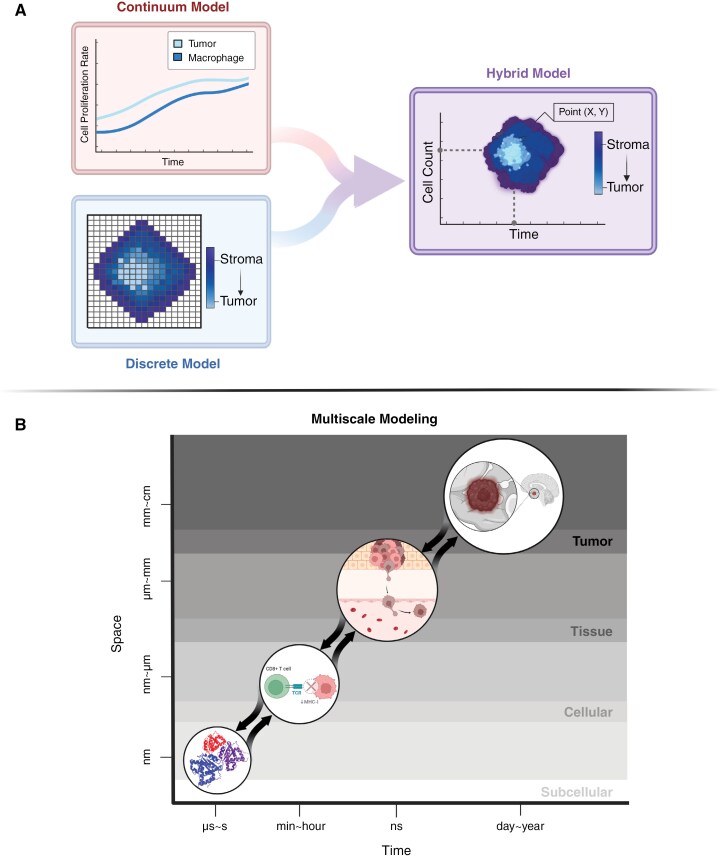
**Approaches to Modeling the Tumor Microenvironment (TME) in pediatric cancers. (A)** *Mathematical Modeling Techniques for TMEs: Continuum Models* treat tumor tissues as a continuous entity, using differential equations to simulate their dynamics. The graph illustrates tumor behavior over time, showing cancer cells (light blue line) and macrophage cells (dark blue line). These models are generally easier to parameterize and interpret compared to other approaches. *Discrete Models* focus on the behavior of individual cells and their interactions with the environment. In the figure, the dark blue areas represent the stroma, while the light blue areas indicate the tumor. This approach is particularly effective for studying cancer progression and cell plasticity at the single-cell level. *Hybrid Models* combine elements of continuum and discrete models to provide a more comprehensive understanding of tumor dynamics. The graph illustrates cell count over time alongside a point-based cell model (X, Y), where dark blue represents the stroma, and light blue depicts the tumor. This hybrid representation captures both tissue-scale dynamics and individual cell interactions. **(B)***Multiscale Modeling*: Multiscale modeling links subcellular, cellular, and tissue-level processes to provide a holistic view of tumor development and the microenvironment. The figure illustrates a progression from a subcellular protein to a CD8 + T cell signaling pathway, then to tissue-level tumor growth, and finally to the macroscopic brain tumor. Created in BioRender. Kumar, K. (2025) https://BioRender.com/t9n35dn.

Mathematical approaches to TME modeling are often divided into categories of either discrete, continuum, or hybrid.^130^ Discrete models evaluate discrete interactions between individual cells, continuum models evaluate tumors through continuous processes like nutrient distribution, and hybrid models combine aspects of both for a more holistic view of the TME (**Figure 3A**).^131^

Agent-based modeling (ABM) is a form of discrete modeling that is used to simulate how specific “agents” behave and interact based on local conditions and system dynamics, where agents are biological entities such as cells or proteins (**Figure 3A**).^132^ Agents are defined by properties and equations that determine their actions based on their own state, nearby entities, and environmental cues.^131^ This approach can be used to understand how conditions of the TME may influence the interactions within it and reveal characteristics such as immune-tumor interactions, immune evasion mechanisms, and drug resistance.^132^ By representing healthy tissue and incorporating extracellular matrix proteins and tumor cells, the impact of factors such as hypoxia and cell density on tumor growth can be simulated.^133^ An ABM developed to evaluate adult glioblastoma response to a combined temozolomide and immune checkpoint blockade regimen found that tumors with high recruitment and infiltration of CD8 + T cells had better treatment responses.^134^ Another ABM combined oncolytic HSV and anti-PD-1 immunotherapy to examine the impact of spatial heterogeneity on dosing strategies for glioblastoma.^135^ The model showed that targeting areas with the highest tumor cell density was more effective than repeated dosing at a single site, underscoring the spatial advantages of computational modeling.^135^

### Continuum Models

Unlike discrete models like ABM, continuum models use differential equations to represent large-scale behaviors such as nutrient diffusion, fluid dynamics, and cell populations as continuous processes (**Figure 3A**).^136^ These models efficiently simulate tumor-immune interactions on a large-scale and offer increased scalability that may be computationally intensive for discrete models.^136^ A 3D continuum mixture model representing the tumor as large tissue systems, simulating extravasation, chemotaxis, and immune cell interactions within the TME was developed.^137^ The study isolated pre-existing immune cell effects and simulated both pro-tumor and anti-tumor environments.^137^ To account for patient-specific tumor characteristics, a multi-objective evolutionary algorithm adjusts radiation dosing based on glioblastoma cell proliferation and diffusion, providing an optimized alternative to standard uniform dosing. A biologically-based continuum model for adult glioblastoma used patient-specific MRI data to simulate untreated tumor growth.^138^ The model created virtual controls, enabling physicians to compare the predicted untreated tumor progression with actual therapeutic outcomes.^138^

While ABM offers insights into cellular dynamics and heterogeneity, they are more computationally intensive due to increased equation complexity.^136^ Conversely, continuum models simplify dynamics using averaged properties, allowing researchers to capture large-scale interactions at lower computational cost.^136^

### Hybrid Models

Hybrid models combine discrete approaches like ABM with continuum models to capture detailed cell-cell interactions while still maintaining large spatial coverage.^136^ Classical hybrid models often use discrete equations to represent tumor agents and continuous differential equations to capture changes in the TME (**Figure 3A**).^136^ One hybrid model integrated continuous tumor growth with a discrete model of tumor-induced angiogenesis to examine immune cell infiltration under hypoxic conditions, identifying a “normalization window” for antiangiogenic therapy.^139^ Another model simulated glioblastoma cell invasion by incorporating cellular phenotypes, the influence of oxygen on cell behavior, and flocking behavior in which cells move collectively in high-density clusters.^140^ Hybrid models also address TME heterogeneity and immunotherapy effects.^136^ A hybrid model showed that anti-angiogenic and anti-mitotic therapies led to a more heterogenous TME and increased glioblastoma invasiveness.^141^ Targeting glioma stem cells (GSCs) and glioma endothelial cells (GECs) was found to be most effective when combined with anti-angiogenic and anti-mitotic therapies, supporting a multi-targeted approach for improved treatment outcomes.^141^ Hybrid models offer 2.4 times greater sensitivity and specificity in identifying tumor variants compared to PDX models, highlighting the potential to significantly improve molecular characterization models and preclinical studies in the pediatric population.^142^

### Multiscale Modeling

Multiscale modeling simulates tumor behavior across multiple biological levels, including subcellular, cellular, and tissue scales (**Figure 3B**).^143^ The subcellular scale encompasses processes like DNA synthesis, the cellular scale captures interactions between cells and the tumor microenvironment, and the tissue scale addresses larger phenomena such as nutrient diffusion and metastasis.^143^ This separation allows researchers to investigate how changes at each level may influence tumor growth and therapeutic response.^143^ A 3D multiscale model of adult glioblastoma growth showed that GSCs receive positive feedback from vascular endothelial cells, promoting tumor growth and VEGF production.^144^ Disrupting this crosstalk reduced tumor volume by 77% and may offer an alternative to traditional antiangiogenic therapy.^144^ A single cell-based multiscale model of brain tumors simulated vascular tumor growth to investigate the role of angiogenesis in drug resistance.^145^ The researchers modeled glioma growth under VEGFR inhibition, revealing how tumors respond to combined EGFR and VEGFR inhibition and identifying co-administration as a strategy to maximize tumor suppression.^145^ A recent multiscale ABM of adult glioblastoma examined how gene mutations and angiogenesis influence tumor growth and drug resistance during tyrosine kinase inhibitor treatment. Although the therapy initially limited tumor growth, early mutations and close proximity to blood vessels eventually promoted tumor proliferation and resistance.^146^

### Computational Models and High-Throughput Data Integration

**Figure 4.**
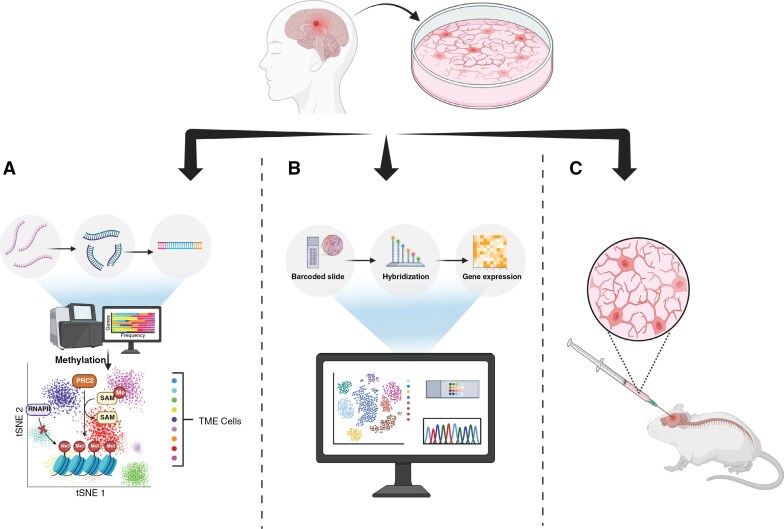
**Techniques for Tumor Modeling and Microenvironment Analysis. (A)** *RNA Sequencing for Tumor Analysis:* Tumor cells are collected from the patient, and RNA is isolated and converted into complementary DNA (cDNA). After adaptor ligation, the cDNA fragments are sequenced using next-generation sequencing technology. The resulting data may then be visualized through t-distributed stochastic neighbor embedding (t-SNE)172, a statistical method that maps high dimensional data into two or three dimensions for analysis. t-SNE shows different colors representing various types of TME cells. This analysis helps identify and categorize the diverse cellular components within the tumor microenvironment. **(B)***Spatial Transcriptomics for Tumor Analysis:* Tumor sections are placed on barcoded slides that capture spatial information of RNA transcripts. Following hybridization, gene expression profiles are generated and analyzed to visualize the spatial organization of cell populations within the TME. **(C)***Xenografting for Tumor Modeling:* Tumor cells are extracted from the patient, cultured in a petri dish, and subsequently injected into mice. This process enables researchers to study tumor progression and potential therapies in vivo. Created in BioRender. Kumar, K. (2025) https://BioRender.com/sbdlr21.

By isolating individual cells to examine gene expression at the single-cell level, scRNA-seq enables researchers to map cellular heterogeneity within the TME and identify tumor subpopulations (**Figure 4A**).^147^ In adult glioblastoma, transcriptomes were compared to those of normal fetal brain cells, revealing that tumor cells originate from a glial progenitor and retain conserved neurodevelopmental gene programs.^148^ Comparative studies showed lower immune cell infiltration and greater myeloid heterogeneity in pediatric gliomas versus adult gliomas, highlighting differences that can be exploited in future immunotherapy.^44^ Multi-omic profiling of H3-K27M DMG tumors across different locations and age groups revealed the presence of OPC-like cells and the absence of neuronal-like cells in both pediatric and adult tumors. MES-like cells were also found to increase with age, correlating with TME differences, such as increased microglia pediatric DMGs and increased macrophages in adult DMGs.^149^

scRNA-seq also holds strong potential for identifying novel targets for future immunotherapies.^150^ One study identified cancer-specific exons as potential targets of CAR T cells in pediatric brain tumors, categorizing the targets as either tier 1 or tier 2 based on minimal or increased expression, respectively.^151^ An analysis of the heterogeneity of immune cells from adult gliomas identified five myeloid subtype gene signatures that function as independent indicators of patient survival and found that phenotype differences among myeloid cells in the TME significantly influence cell-to-cell communication.^150^ Applications of scRNA-seq have further expanded into lineage tracing, leading to the development of computational systems like ClonMapper, which integrates scRNA-seq with DNA barcoding to characterize clones within heterogeneous cancer populations and identify high survivorship clones that dominate post-chemotherapy treatment.^152^ scRNA-seq has the ability to understand TME composition, tracking tumor evolution, and identifying pathways critical for tumor cell survival.^153^

While scRNA seq provides insight into tumor heterogeneity, it is limited in its ability to capture the complex spatial organization of a tumor and the TME.^154^ Spatial transcriptomics has recently emerged as a powerful method to address this by preserving the spatial architecture of a tumor sample while profiling gene expression (**Figure 4B**).^154^ Researchers can perform spatial transcriptomic profiling across all genes or apply targeted gene panels associated with specific cell types of biological pathways to improve tumor-specific analysis and immune profiling.^155^ Glioma cells are known to cluster into spatially distinct niches with unique cellular components that influence tumor growth and therapeutic response, making spatial characterization a valuable addition that is missing in traditional scRNA-seq analyses.^156^ Spatial profiling of DMG and glioblastoma identified four distinct spatial niches including hypoxic niche, vascular niche, invasive edge, and tumor core that may inform region-specific therapies.^156^ Additionally, researchers found enrichment of radial-glial-like cells in the invasive niche in both DMG and glioblastoma samples, supporting their potential role in tumor invasion.^156^ Spatial transcriptomics of pHGGs revealed dominance of the tumor immune microenvironment by immunosuppressive myeloid cells with reduced CD8 T cell proximity, offering novel insights into immune cell spatial dynamics.^157^ Spatial transcriptomic analysis of pHGGs confirmed their immunologically cold nature by demonstrating < 0.5% T cell detection within tissue samples.^158^ By preserving spatial context, the study revealed age-specific clustering and distinct proportions of myeloid cell types compared to adult HGGs, enabling high-resolution mapping of immune cell niches within and beyond the tumor core.^158^

Mathematical and computational modeling play a crucial role in advancing our understanding of tumor biology and subcellular processes. Integrating computational data with in vivo models allows researchers to validate computational findings and confirm therapeutic targets identified through these models.

### Patient-Derived Xenograft (PDX) and Genetically Engineered Models (GEM)

PDX models are considered one of the most effective preclinical models for evaluating anti-cancer therapies and drug-resistance mechanisms.^159^ A key feature of PDX models is their ability to preserve the unique heterogeneity of the TME.^159^ To develop PDX models, a sample of either a primary or metastatic tumors is implanted into immunodeficient mice, preserving the morphology and architecture of the original tumor (**Figure 4C**). To prevent rejection, PDX models are usually created with immunocompromised mice.^160^

PDX models are a widely utilized tool to understand tumor biology and tumor therapy due to its ability to precisely preserve the structure of the TME.^161^ Specifically, their ability to maintain high tumor heterogeneity, gene expression, and mutations makes it ideal for biomarker evaluation.^159^ Many studies have highlighted its efficacy in pediatric brain tumor research.^162^ PDX models of pediatric DMG have been generated from stereotactic biopsies taken at diagnosis, successfully capturing key features of DMG, including its infiltrative nature, lack of tumor mass, and expression of the characteristic H3-K27M mutation.^163^ High-throughput screening in PDX models of pHGGs found that in vitro results could predict in vivo responses to PI3K/mTOR and MEK pathway inhibitors.^164^

The Children’s Brain Tumor Network has developed a library of over 150 preclinical models, including cell lines and PDXs, to support pediatric brain tumor research.^165^ These models contain molecular data, including whole-genome sequencing and RNA-seq, and have been utilized by over 50 research projects internationally.^165^ While PDX models offer insight into tumor heterogeneity and therapeutic response, their use in immunocompromised mice limits their ability to model the immune microenvironment and tumor-immune interactions, which are both critical components of the TME.^166^ Newer approaches include humanized mouse models, which use human cell-engrafted immunodeficient mice to simulate human immune responses, and syngeneic models, which implant tumors into immunocompetent mice to better assess tumor-immune interactions and the tumor microenvironment.^161^

Genetically engineered models (GEM) offer critical insight into pediatric glioma genesis by deliberate induction of tumorigenic genomic alterations within an immunocompetent host, allowing researchers to study tumor-immune cell interactions and the influence of genetic drivers on the TME.^167^ One widely used system is the replication-competent avian sarcoma-leukosis virus long terminal repeat with splice acceptor/tumor virus A (RCAS/tv-a) model in which the RCAS virus selectively delivers oncogenes into cells expressing the tv-a receptor.^127^ The RCAS vector’s limited penetration enables a low number of cells to uptake the target gene, enabling a close representation of events early in tumorigenesis.^127^ In utero electroporation represents another approach in which oncogenic plasmids are delivered into the brains of developing mouse embryos and electroporated into neural progenitor cells, allowing for the study of glioma formation during normal brain development.^127^ Another model widely used for pediatric gliomas is the Sleeping Beauty (SB) transposon delivery model in which DNA plasmids containing oncogenes are injected into cells using the SB transposase, allowing for controlled gene expression and identification of genetic drivers.^168^

## Discussion

The landscape of immunotherapy for pediatric gliomas is evolving based on the unique biological and immunological features that set them apart from their adult counterparts. Pediatric tumors exhibit an immunologically “cold” TME characterized by a low mutational burden, a limited presence of neoantigens, and decreased immune cell infiltration. These properties diminish the efficacy of immunotherapies that leverage immune functions, such as checkpoint inhibitors or CAR T-cell therapy. Cellular components within the TME also differ in pediatric populations. TAMs play divergent roles in pediatric versus adult gliomas, and while adult gliomas frequently exploit PD-L1-mediated immune evasion, PD-L1 expression is low in pediatric gliomas. Furthermore, distinct epigenetic profiles, such as the H3K27M mutation and differing progenitor pathways, underscore the developmental origins of pediatric gliomas.

These findings question reliance on adult glioma research and highlight the need for pediatric-specific models and trials to develop age-appropriate immunotherapies. Barriers to conducting pediatric-specific clinical trials include recruitment difficulties, limited cohort sizes, and the need for child-specific tumor banks. Integrating high-throughput -omic technologies and advanced computational modeling presents a promising alternative to address these gaps. We emphasize these methods to highlight their underutilization in pediatric gliomas, with the hope that future researchers will adopt them. Among the current immunotherapy clinical trials in pediatric brain tumors, oncolytic viruses such as DNX-2401 and PVSRIPO have demonstrated promising early results and further advancement in the clinical trials process as compared to CAR T and neoantigen vaccine therapies. This suggests that viral-based therapies may be uniquely equipped to penetrate the immunosuppressive TME of pediatric gliomas. Computational modeling can be leveraged to further refine delivery methods, optimize dosing schedules, and predict the synergistic effects of combining across therapy types. Barriers to current immunotherapies, including penetrating the blood–brain barrier and the lack of neoantigen presentation can be overcome by creating spatial visualizations of the TME and modeling discrete cellular characters to identify therapeutic targets and weaknesses in the TMEs defenses that can be exploited. These strategies provide researchers with ways to bypass clinical trial limitations while advancing pediatric tumor therapies. Computational virtual models of pediatric brain tumors, validated with clinical imaging, could serve as surrogates for therapeutic testing.

While this study provides insights into the potential of computational modeling for pediatric gliomas, there are some limitations. The underutilization of these models in pediatric gliomas has limited the scope of current findings by relying on adult glioma data and data extrapolated from non-CNS tumors. Lack of integration between models and clinical data hinders translation of predictions into effective therapies. Another important constraint is the limited number of pediatric-specific clinical trials and datasets, which may restrict the generalizability of our findings across diverse patient populations. Future research efforts should incorporate more pediatric-specific datasets and developing computational models that integrate age-specific tumor biology. Despite limitations, this article advances understanding of the TME in pediatric gliomas and underscores the potential of computational modeling. Integrating *in silico* and in vivo approaches may enable patient-specific interventions and guide age-specific therapies to improve outcomes.
